# Potential Downstream Target Genes of Aberrant ETS Transcription Factors Are Differentially Affected in Ewing’s Sarcoma and Prostate Carcinoma

**DOI:** 10.1371/journal.pone.0049819

**Published:** 2012-11-19

**Authors:** Maria J. Camões, Paula Paulo, Franclim R. Ribeiro, João D. Barros-Silva, Mafalda Almeida, Vera L. Costa, Nuno Cerveira, Rolf I. Skotheim, Ragnhild A. Lothe, Rui Henrique, Carmen Jerónimo, Manuel R. Teixeira

**Affiliations:** 1 Department of Genetics, Portuguese Oncology Institute-Porto, Porto, Portugal; 2 Cancer Genetics Group, Research Centre of the Portuguese Oncology Institute-Porto, Porto, Portugal; 3 Cancer Epigenetics Group, Research Centre of The Portuguese Oncology Institute, Porto, Portugal; 4 Department of Cancer Prevention, Institute for Cancer Research, Norwegian Radium Hospital, Oslo University Hospital, Oslo, Norway; 5 Centre for Cancer Biomedicine, Faculty of Medicine, University of Oslo, Oslo, Norway; 6 Department of Pathology, Portuguese Oncology Institute-Porto, Porto, Portugal; 7 Department of Pathology and Molecular Immunology, Institute of Biomedical Sciences Abel Salazar (ICBAS), University of Porto, Porto, Portugal; Tulane University School of Medicine, United States of America

## Abstract

*FLI1* and *ERG*, the major ETS transcription factors involved in rearrangements in the Ewing’s sarcoma family of tumors (ESFT) and in prostate carcinomas (PCa), respectively, belong to the same subfamily, having 98% sequence identity in the DNA binding domain. We therefore decided to investigate whether the aberrant transcription factors in both malignancies have some common downstream targets. We crossed a publicly available list of all putative EWSR1-FLI1 target genes in ESFT with our microarray expression data on 24 PCa and 6 non-malignant prostate tissues (NPT) and choose four genes among the top-most differentially expressed between PCa with (PCa *ERG+*) and without (PCa ETS-) ETS fusion genes (*HIST1H4L*, *KCNN2, ECRG4* and *LDOC1*), as well as four well-validated direct targets of the EWSR1-FLI1 chimeric protein in ESFT (*NR0B1*, *CAV1*, *IGFBP3* and *TGFBR2*). Using quantitative expression analysis in 16 ESFT and seven alveolar rhabdomyosarcomas (ARMS), we were able to validate the four genes previously described as direct targets of the EWSR1-FLI1 oncoprotein, showing overexpression of *CAV1* and *NR0B1* and underexpression of *IGFBP3* and *TGFBR2* in ESFT as compared to ARMS. Although none of these four genes showed significant expression differences between PCa *ERG*+ and PCa ETS-, *CAV1, IGFBP3* and *TGFBR2* were less expressed in PCa in an independent series of 56 PCa and 15 NPT, as also observed for *ECRG4* and *LDOC1*, suggesting a role in prostate carcinogenesis in general. On the other hand, we demonstrate for the first time that both *HIST1H4L* and *KCNN2* are significantly overexpressed in PCa *ERG+* and that ERG binds to the promoter of these genes. Conversely, *KCNN2* was found underexpressed in ESFT relative to ARMS, suggesting that the EWSR1-ETS oncoprotein may have the opposite effect of ERG rearrangements in PCa. We conclude that aberrant ETS transcription factors modulate target genes differently in ESFT and PCa.

## Introduction

The involvement of ETS genes in cancer was first demonstrated by the presence of the oncogene v-ets as part of the gag-myb-ets transforming fusion protein of an avian retrovirus, E26 [1]. Their importance in human carcinogenesis is supported by the observations that ETS genes are implicated in chromosomal translocations, giving rise to fusion proteins that play an important role in the genesis of several hematological malignances, soft tissue tumors and carcinomas [2]. The ETS family of transcription factors is one of the largest families of transcription regulators (27 members in the human genome), and plays an important role in diverse biological processes, including cell proliferation, apoptosis, differentiation, lymphoid and myeloid cell development, angiogenesis and invasiveness [3]–[4]. It is characterized by an 85 amino acidic, highly conserved, DNA binding domain (known as ETS domain), which displays sequence specific binding to purine-rich DNA sequences containing a 5′-GGAA/T-3′ core sequence [5]–[6].

The Ewing’s sarcoma family of tumors (ESFT) serves as a paradigm for the entire class of ETS-related tumors, since more than 99% of the cases harbor translocations involving ETS genes and *EWSR1* [7]. In 85% of the cases, the ESFT harbors a t(11;22)(q24;q12) chromosomal translocation, resulting in a fusion of the amino terminus of the *EWSR1* gene to the carboxyl terminus (containing the DNA binding domain) of *FLI1*. Fusions between *EWSR1* and other ETS genes, namely *ERG* (10%) and *ETV1*, *ETV4*, or *FEV* (<5%), are alternative pathogenetic mechanisms in ESFT [7]. Prostate cancer (PCa) is the most recent ETS-related neoplasia [8], with the *TMPRSS2-ERG* fusion gene being reported in about 50% of the cases [8]–[11]. Other, less common gene fusions (1–10%), involve additional ETS family members, such as *ETV1*, *ETV4*, *ETV5*, and *FLI1* [12]–[14]. In both ESFT and PCa these ETS chimeric genes function as aberrant transcription factors, having a pivotal role in promoting transformation and oncogenesis. This hypothesis is consistent with experiments showing that *EWSR1-FLI1* knockdown is correlated with decreased cell invasion and increased apoptosis [15]–[16] and with reports showing that overexpression of *ERG* and *ETV1* in benign prostate cells induces a transcriptional program associated with invasion [17]–[18].

Identifying the target genes of the ETS fusion genes is crucial to understand the oncogenic pathways of the ETS-positive malignancies and some of them may turn out to be more amenable to targeted therapy than the chimeric/truncated transcription factors themselves. Whereas several target genes relevant for ESFT have been uncovered [19]–[20], the search for the downstream effectors of aberrant ETS transcription factors in PCa is still in its infancy [21]–[22]. The major ETS genes involved in rearrangements in ESFT and PCa, *FLI1* and *ERG*, respectively, belong to the same subfamily, have 98% sequence identity in the DNA binding domain [23]–[24], and have been found rearranged in both neoplasias [7]–[8], [13]. In order to investigate whether these ETS fusion genes have some common downstream targets, we crossed a publicly available list of all putative *EWSR1-FLI1* direct target genes in ESFT (obtained by chromatin immunoprecipitation coupled with DNA microarrays) [20] with our microarray expression data on PCa with and without *ERG* rearrangements [25] and validated the findings in an independent series of PCa and ESFT.

## Materials and Methods

### Ethics Statement

This study was approved by the institutional review board (Comissão de Ética para a Saúde). Written informed consent was obtained for all participants.

### Selection of Candidate ETS Target Genes

To select the ETS candidate target genes, we started from the list of 874 genes shown by Gangwal and colleagues [20] to be bound by EWSR1-FLI1, the main ETS fusion protein involved in ESFT tumorigenesis. To accomplish this task, they used a combined approach that included chromatin immunoprecipitation and microarray technology. Based on that list, we then used our whole genome expression data on PCa and non-malignant prostatic tissues (NPT) [25], to find out how many of those genes were relevant in prostate carcinogenesis. The genome-wide RNA expression analysis included 6 NPT and 24 PCa: 16 with *ERG* fusion genes (PCa *ERG+*) and 8 without ETS rearrangements (PCa ETS-) as determined by FISH and reverse-transcription-PCR (RT-PCR) [9], [25]. Then the following selection criteria were applied: a) the gene expression had to be at least 2-fold higher or 1.5-fold lower in PCa harboring *ERG* fusion genes compared to those negative for ETS rearrangements; b) the expression ratio between ETS negative carcinomas and NPT had to be similar (between 0.9 and 1.1).

Four well validated direct targets of the EWSR1-FLI1 chimeric protein in ESFT were selected based on a literature survey. These included the two upregulated genes *CAV1* [26] and *NR0B1* [27] and the two downregulated genes *IGFBP3* [16] and *TGFBR2* [28].

### Prostate Cancer and Non-malignant Tissue Specimens

Fifty-six PCa samples were selected from a pool of 200 patients with clinically localized prostate adenocarcinoma consecutively diagnosed and treated with radical prostatectomy at the Portuguese Oncology Institute – Porto (IPO-Porto), Portugal [13]. These samples were chosen in order to represent different molecular subtypes of prostate cancer, as previously classified, and included: 24 samples with *ERG* rearrangements (PCa *ERG*+), 12 with other ETS rearrangements (PCa oETS+, which include rearrangements with ETS members of the PEA3 subfamily – *ETV1*, *ETV4* and *ETV5* [24]) and 20 without ETS rearrangements (PCa ETS-). For control purposes, 15 NPT were collected from cystoprostatectomy specimens of bladder cancer patients who did not harbor simultaneous prostate carcinoma.

### Ewing’s Sarcoma and Alveolar Rhabdomyosarcoma Samples

Sixteen samples of ESFT were used. RT-PCR was performed to detect the respective fusion transcripts [29] as part of routine molecular diagnosis at the Department of Genetics of IPO-Porto. Fourteen out of sixteen (88%) samples presented the *EWSR1-FLI1* fusion transcript and the remaining two (12%) had the *EWSR1-ERG* chimeric protein. Because the cell of origin of ESFT is not known, we used as control seven alveolar rhabdomyosarcomas (ARMS), which are also small blue round cell tumors but do not express ETS chimeric proteins; instead, they are characterized by the specific translocation t(2;13)(q35;q14) or its variant t(1;13)(p36;q14) giving rise to the fusion genes *PAX3-FKHR* or *PAX7-FKHR,* respectively [30]. Using RT-PCR as part of routine molecular diagnosis in our department [31], the *PAX3-FKHR* was detected in four (57%) samples and the remaining three (43%) had the *PAX7-FKHR* fusion transcript. RNA samples from the 16 ESFT and the seven ARMS were used for the target gene analyses.

### Prostate Cell Lines

LNCaP cells were acquired from the German Resource Centre for Biological Material (DSMZ, Braunschweig, Germany) and 22Rv1 cells were kindly provided by Dr David Sidransky from the Johns Hopkins University School of Medicine. Both cell lines were cultured under the recommended conditions, being karyotyped by G-banding for validation purposes and tested for Mycoplasma spp. Contamination (PCR Mycoplasma Detection Set; Clontech Laboratories, Saint-Germain-en-Laye, France).

### RNA Extraction and cDNA Synthesis

Total cellular RNA was extracted from the prostate tissue samples using the TRIzol® reagent combined with the Purelink™ RNA Mini Kit purification columns (Invitrogen by Life Technologies, Carlsbad, CA), as previously described [25]. Subsequently, 200 ng of RNA were converted into cDNA using the TransPlex Whole Transcriptome Amplification Kit (Sigma-Aldrich, St. Louis, MO), according to the manufacturer’s instructions. For total RNA extraction from cell lines, the TRIzol® reagent was used, following the manufacturer’s recommendations. cDNA was obtained from 500 ng of RNA using random hexamer primers and the H-minus RevertAid cDNA synthesis kit (Fermentas, Ontario, Canada), according to the manufacturer’s instructions.

### DNA Extraction and Bisulfite Treatment

To assess whether decreased gene expression was associated with DNA methylation, DNA was extracted from prostate tissue samples and from cell lines by the phenol-chloroform method [32], and subsequently subjected to sodium bisulfite conversion using the EZ DNA Methylation-Gold™ Kit (Zymo Research, Orange, CA), according to the manufacturer’s protocol. CpGenome™ Universal Methylated DNA (Millipore, Billerica, MA) and CpGenome™ Universal Unmethylated DNA (Millipore) were also bisulfite-modified to serve as positive and negative controls, respectively.

### Cell Line Treatment with 5-aza-2′deoxycytidine (DAC)

To evaluate whether promoter methylation of *CAV1*, *IGFBP3* and *ECRG4* was associated with decreased transcript expression in PCa, we treated LNCaP and 22Rv1 prostate cancer cell lines (the first harboring an *ETV1* rearrangement and the second without known ETS rearrangements) with 1 µM of the DNA methyltransferases inhibitor 5-aza-2′deoxycytidine (DAC; Sigma-Aldrich), as previously described [33]. After 72 hours of treatment, DNA and RNA were extracted as described above.

### Quantitative RT-PCR (qRT-PCR)

In order to determine the relative expression levels of selected genes, qRT-PCR was performed. Primers and probes for the selected genes and the endogenous control (glucoronidase beta, *GUSB*) were acquired as pre-developed TaqMan® Gene Expression Assays from Applied Biosystems (by LifeTechnologies, Foster City, CA) (Supplementary Table S1). *GUSB* gene was used for normalization of the expression levels of the selected genes. All samples were run in triplicate and multiple negative controls were included in each plate. Relative expression values were obtained by the comparative Ct method [34].

### Methylation-specific PCR (MSP) and Quantitative MSP (qMSP)

To confirm the presence of a CpG island in the promoter region of the genes of interest, their RefSeqs were obtained from the USCS Genome Browser Database (http://genome.ucsc.edu/), including the 2 Kb sequence upstream of the first exon, and these were subsequently analyzed *in silico* using CpG Island Searcher software, according to the algorithm described by Takai and Jones (2002) [35]. The primers’ sequences for *CAV1*, *IGFBP3*, and *LDOC1* have been published elsewhere [36]–[38] and the primers’ sequences for *TGFBR2* and *ECRG4* are shown in Supplementary Table S1, all being acquired from Metabion (Martinsried, Germany). MSP assays were carried on prostate samples using 2 µL of template modified-DNA in a 20 µL PCR reaction containing 0.2 mM of dNTPs mix (Fermentas, Ontario, Canada), 0.25 µM of each primer and 0.5 U of DyNAzyme™ II Hot Start (Finnzymes) in 1x DyNAzyme™ II Hot Start Reaction Buffer (Finnzymes, Vantaa, Finland). PCR was then performed according to the DyNAzyme™ II Hot Start manufacturer’s conditions. Considering the limited amount of bisulfite-treated DNA available for the MSP analysis, samples were selected according to the lowest expression for each gene (14 for *ECRG4*, 10 for *CAV1*, eight for *IGFBP3* and *LDOC1* and seven for *TGFBR2*) (Supplementary Table S2).

For qMSP on DAC-treated cell lines, 2 µL of bisulfite modified-DNA were amplified with 0.25 µM of each primer in 1× Power SYBR® Green PCR Master Mix (Applied Biosystems). β-Actin (*ACTB*, Supplementary Table S1) was used as an internal reference gene to normalize for DNA input and the qMSP reaction was performed as previously described [33].

### Chromatin Immunoprecipitation (ChIP) and Quantitative PCR (qPCR)

We used VCaP cells and the rabbit anti-ERG monoclonal antibody (Epitomics, Burlingame, CA) to detect ERG binding to the promoter of *HIST1H4L* and *KCNN2*, as previously described [25]. Briefly, 2×10^6^ cells were used for each immunoprecipitation with the EZ-Magna ChIP™ G kit (Millipore), following manufacturer’s instructions [39]. To select for putative ETS binding sequences in the promoter regions, a bioinformatic survey of the 10 kb sequence upstream of the translation start site was conducted using ConSite [40]. Three promoter regions of *HIST1H4L* (−454, −728 and −2266), each containing two putative ETS binding sequences, and three promoter regions of *KCNN2* (−1442, −1833 and −4083), the first two containing one putative ETS binding sequence and the last containing three, were selected for qPCR analysis of the ERG-immunoprecipitated chromatin. Primers were designed using the Primer3 online software and acquired from Metabion. Primers for a negative control region were also included to correct for unspecific binding (Supplementary Table S1) [41]. qPCR was performed using Power SYBR® Green (Applied Biosystems), according to the manufacturer’s recommendations. Serial dilutions of the input fraction were used to calculate primers’ efficiency. Results are shown as a fold enrichment of ERG bound chromatin relative to IgG and corrected to the negative control region [42].

### Statistical Analysis

Differences in relative expression values of each gene in different groups were assessed by the Kruskall-Wallis non-parametric test, followed by pair-wise comparisons using the Mann-Whitney non-parametric test. The Chi-square test was used to assess the statistical significance of the differences in the frequency of methylation between NPT and PCa samples and a t-test was applied to qPCR and qMSP data. A *p-*value below 0.05 was considered statistically significant. The statistical analyses were performed using the Statistical Package for Social Sciences software, version 15.0 (SPSS Inc., Chicago, IL).

## Results

### Microarray Expression Data and Candidate Target Gene Selection

After crosschecking the list of *EWSR1*-*FLI1* target genes in ESFT [20] with our microarray expression data on PCa and NPT, and applying the aforementioned selection criteria, seven potential ETS target genes emerged. Two genes were overexpressed in PCa with *ERG* fusion genes, namely *HIST1H4L* and *KCNN2*, and were chosen for validation. Five genes were underexpressed in PCa with *ERG* fusion genes, namely *ABCD1, ECRG4*, *KCNMA1*, *LDOC1 and SLC7A4*. *ECRG4* and *LDOC1* were selected for further analysis based on their putative function as tumor suppressor genes in other cancer types [43]–[44].

The expression of the selected target genes in Ewing’s sarcoma (*CAV1*, *NR0B1*, *IGFBP3* and *TGFBR2*), together with the expression of *HIST1H4L*, *KCNN2*, *ECRG4* and *LDOC1*, was then validated in an independent series of PCa with and without ETS gene fusions, as well as in a series of ESFT and ARMS.

### *CAV1* Relative Expression

**Figure 1:**
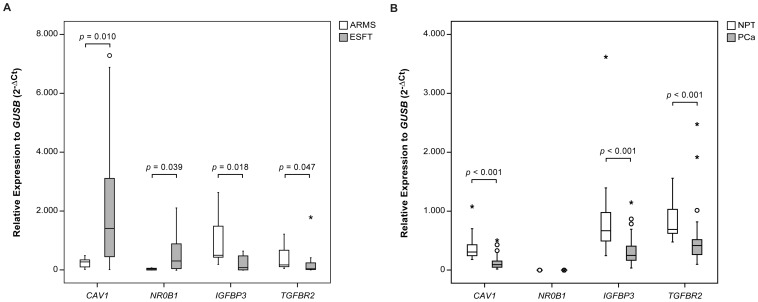
Box-plot representation of the qRT-PCR data for the four genes described as EWSR1-FLI1 targets (*CAV1*, *NR0B1*, *IGFBP3* and *TGFBR2*). **A)** ESFT *versus* ARMS samples; **B)** PCa *versus* NPT samples. A *p* value is shown whenever the differences in each two group comparison reach significance (*p*<0.05).

**Figure 2:**
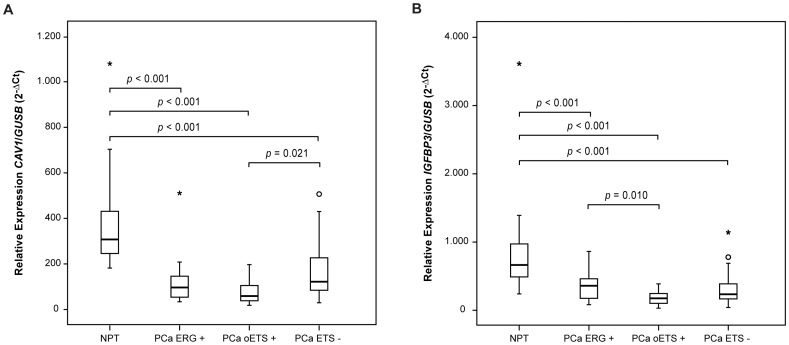
Box-plot distribution of *CAV1* and *IGFBP3* expression in PCa sample subgroups. **A)** *CAV1* expression; **B)** *IGFBP3* expression. A *p* value is shown whenever the differences in each two group comparison reach significance (*p*<0.05).

*CAV1* was significantly overexpressed in ESFT when compared to ARMS, showing a median 4.9 fold increase (Figure 1A). On the other hand, *CAV1* was significantly underexpressed in PCa ETS+ when compared to PCa ETS−, presenting a median 1.5 fold decrease (not shown). Although there was no significant difference in *CAV1* expression between PCa *ERG+* and PCa ETS−, *CAV1* expression in PCa oETS+ was significantly lower when compared to PCa ETS−, with a median 5.5 fold decrease (Figure 2A). *CAV1* expression was significantly lower (3.3 fold decrease) in PCa in general when compared to NPT (Figure 1B).

### *NR0B1* Relative Expression

*NR0B1* relative expression was significantly higher in ESFT when compared to ARMS, showing a median 8.3 fold increase (Figure 1A). On the contrary, *NR0B1* was poorly expressed in PCa and NPT (Figure 1B) and there were no significant differences in relative expression between these groups or among different molecular subgroups of PCa (not shown).

### *IGFBP3* Relative Expression

*IGFBP3* expression was significantly decreased in ESFT when compared to ARMS, exhibiting a median 7.7 fold decrease (Figure 1A). On the other hand, *IGFBP3* relative expression did not show significant differences in different molecular subgroups of PCa, except between PCa *ERG*+ and PCa oETS+ (the last group presenting a median 2 fold decrease expression level; Figure 2B). Globally, *IGFBP3* was significantly underexpressed in PCa when compared to NPT, presenting a median 2.7 fold decrease (Figure 1B).

### *TGFBR2* Relative Expression

*TGFBR2* was significantly underexpressed in ESFT when compared to ARMS, showing a median 3.7 fold decrease (Figure 1A). In contrast, *TGFBR2* expression did not show significant differences among the three molecular subgroups of PCa (not shown). However, when considering PCa as a sole entity we observed that *TGFBR2* expression was significantly lower when compared to NPT (Figure 1B).

### *HIST1H4L* Relative Expression

**Figure 3:**
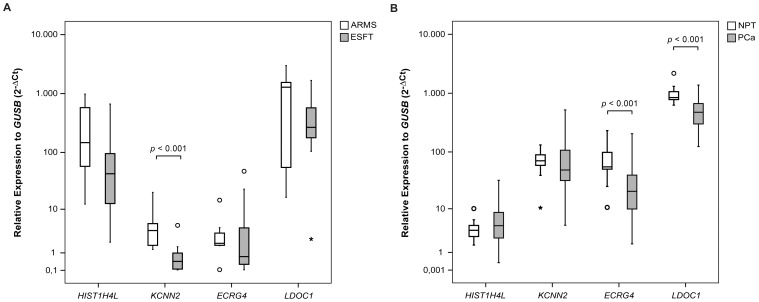
Box-plot representation of the qRT-PCR data for the four genes described as EWSR1-FLI1 targets and associated with PCa samples harboring *ERG* rearrangements (*HIST1H4L*, *KCNN2*, *ECRG4* and *LDOC1*). **A)** ESFT *versus* ARMS samples; **B)** PCa samples *versus* NPT samples. A *p* value is shown whenever the differences in each two group comparison reach significance (*p*<0.05).

**Figure 4:**
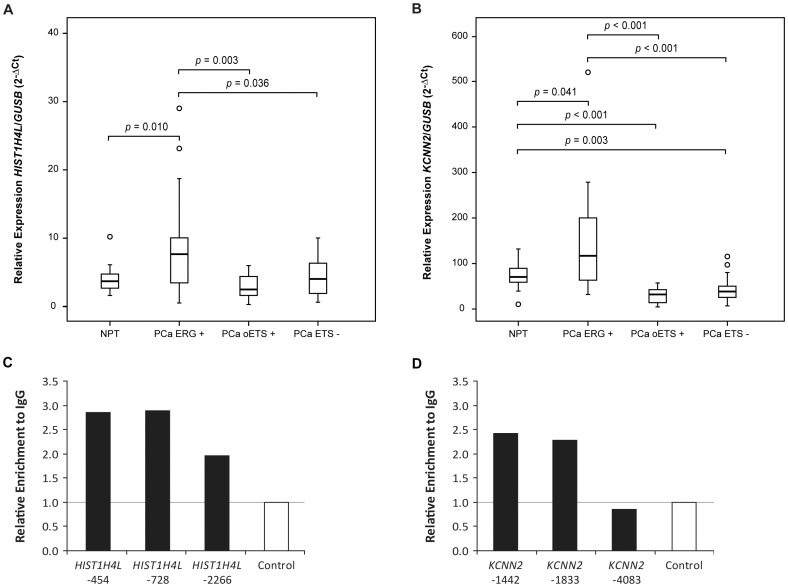
Analyses of *HIST1H4L* and *KCNN2* expression and their regulation by ERG in PCa samples harboring *ERG* rearrangements. **A)** and **B)** Box-plot distribution of *HIST1H4L* and *KCNN2* expression in PCa sample subgroups, respectively. A *p* value is shown whenever the differences in each two group comparison reach significance (*p*<0.05). **C)** and **D)** qPCR of ERG-immunoprecipitated chromatin from VCaP cells showing ERG binding to three regions of the *HIST1H4L* promoter and to two regions of the *KCNN2* promoter, respectively.

*HIST1H4L* relative expression was not significantly different between ESFT and ARMS (Figure 3A). On the other hand, although the expression of *HIST1H4L* was not significantly different between PCa in general and NPT (Figure 3B), PCa *ERG+* presented higher *HIST1H4L* expression levels when compared to PCa oETS+ (median 3.0 fold increase), PCa ETS− (median 1.9 fold increase) and NPT (median 2.1 fold increase) (Figure 4A).

### *KCNN2* Relative Expression

*KCNN2* was poorly expressed in ESFT and ARMS, but it was significantly underexpressed in the former when compared to the later, showing a median 9.4 fold decrease (Figure 3A). On the other hand, although the expression of *KCNN2* was not significantly different between PCa in general and NPT (Figure 3B), the relative expression of *KCNN2* in PCa ETS+ was significantly higher when compared to PCa ETS− (p = 0.011), showing a median 1.7 fold increase (not shown). This significant overexpression was found when comparing PCa *ERG+* with either PCa oETS+ or PCa ETS−, with a median 3.7 and 3.0 fold increase, respectively, but not between PCa oETS+ and PCa ETS− (Figure 4B). *KCNN2* was also significantly overexpressed in PCa *ERG+* when compared to NPT, showing a median 1.7 fold increase, but was significantly underexpressed in PCa oETS+ and PCa ETS− when compared to NPT, displaying a median 2.2 and 1.8 fold decrease, respectively (Figure 4B).

### *ECRG4* Relative Expression

*ECRG4* relative expression was not significantly different between ESFT and ARMS (Figure 3A). Similarly, there were no significant differences in *ECRG4* relative expression among the different molecular subgroups of PCa (not shown). However, *ECRG4* expression was significantly decreased (2.7 fold) in PCa when compared to NPT (Figure 3B).

### *LDOC1* Relative Expression

There was no significant difference in *LDOC1* expression between ESFT and ARMS (Figure 3A). Likewise, *LDOC1* expression did not present significant differences among the different molecular subgroups of PCa (not shown). Nonetheless, *LDOC1* was underexpressed (1.8 fold decrease) in PCa in general when compared to NPT (Figure 3B).

### Promoter Hypermethylation and Downregulation of *CAV1*, *IGFBP3* and *ECRG4* in PCa

The promoter methylation status of *CAV1*, *IGFBP3*, *TGFBR2*, *ECRG4* and *LDOC1* was evaluated in prostate tissue samples (Supplementary Table S2). Although we were not able to detect differences among PCa subgroups, overall, higher promoter methylation frequencies of *CAV1*, *IGFBP3* and *ECRG4* were found in PCa compared to NPT (*p* = 0.010 for *CAV1*, *p*<0.001 for *IGFBP3* and *p* = 0.008 for *ECRG4*). No methylation was detected at the *TGFBR2* and *LDOC1* promoters in prostate tumor samples. DAC-treatment of the *ETV1* rearrangement-positive cell line LNCaP resulted in decreased methylation of *CAV1* promoter and *de novo CAV1* expression, although the difference did not reach statistical significance (*p* = 0.07; Supplementary Figure S1). A slight increase in *IGFBP3* expression was also observed in LNCaP cells after DAC treatment, although not statistically significant (*p* = 0.15; data not shown). The ETS-negative cell line 22Rv1 showed basal expression of *CAV1* and *IGFBP3,* which did not change after DAC treatment. *ECRG4* was not expressed in both cell lines and DAC treatment was not sufficient to induce *de novo ECRG4* expression (data not shown).

### ERG Binds to *HIST1H4L* and *KCNN2* Promoter Regions

Using ChIP of VCaP cells, we were able to detect ERG binding to the three regions tested for the *HIST1H4L* promoter (−454, −728 and −2266) and to two regions of the *KCNN2* promoter (−1442 and −1833) (Figures 4C and 4D).

## Discussion

The ETS family of transcription factors is one of the largest involved in the regulation of a variety of different genes that play key roles in proliferation, apoptosis, differentiation, hematopoiesis, metastasis, tissue remodeling, angiogenesis and transformation [3]–[4]. Identification of the target genes for normal and oncogenic ETS genes may lead to a better understanding of the mechanisms underlying malignant transformation. All ETS family members bind to 5′-GGAA/T-3′ DNA sequences and ETS target genes may be identified upon the presence of a functional binding site in their regulatory regions [5]–[6]. The crucial role of ETS chimeric proteins in the development of ESFT is well documented [45], and although it has been shown that EWSR1-FLI1 and EWSR1-ERG bind ETS sequences *in vitro* with similar specificities and affinities as the wild-type transcription factors [46]–[47], it has recently been shown that EWSR1-ETS chimeric proteins induce chromatin structure alterations that lead to transcription dysregulation [48]. Contrary to ESFT, in PCa the most common ETS fusion member is *ERG* (about 90% of the fusion positive cases), and only 1–10% of the cases have fusion genes involving other ETS members, namely, *ETV1*, *ETV4* and *ETV5* (which cluster in the PEA3 subfamily), and *FLI1* (which clusters with *ERG* in the ERG subfamily) [8], [12]–[14], [23]. The ETS domain of the PEA3 subfamily displays 60% homology with the ERG subfamily, but there is no significant homology outside the ETS domain as indicated by the presence of a PNT domain in the ERG subfamily but not in the PEA3 subfamily [6], [23]. Recently, we showed that while some genes are specifically and differentially expressed between PCa harboring *ERG* or *ETV1* rearrangements, others are commonly dysregulated between these tumor molecular subtypes and PCa without ETS rearrangements, with *ETV4* and *ETV5* positive tumors clustering together with those with *ETV1* rearrangement [33]. As *FLI1* and *ERG* belong to the same subfamily and share 98% of sequence homology in the DNA binding domain [23]–[24], we questioned whether in a different cell background they would show dysregulation of the expression of the same genes.

We started by analyzing the expression of four well-validated targets of the EWSR1-FLI1 oncoprotein in ESFT and we used ARMS for control purposes. We validated the dysregulation in the expression of four genes previously described as direct targets of the EWSR1-FLI1 oncoprotein in ESFT, showing overexpression of *CAV1* and *NR0B1* and underexpression of *IGFBP3* and *TGFBR2* [16], [26]–[28]. We then evaluated the expression of these genes in PCa with and without ETS rearrangements. None of these genes showed significant expression differences between PCa *ERG*+ and PCa ETS−, suggesting that *ERG* proteins do not regulate their expression in this tumor type. However, the expression of *CAV1, IGFBP3* and *TGFBR2* is decreased in PCa in general, suggesting a role in prostate carcinogenesis. Our data suggest that regulation of *CAV1* expression may be, at least in part, controlled by promoter methylation, which has also been reported by others [49]. Although we found decreased *CAV1* expression especially in PCa oETS+ and the *ETV1-*positive LNCaP cell line showed increased expression of *CAV1* after DAC treatment, the methylation status of the *CAV1* promoter in PCa oETS+ samples was heterogeneous, while in other tumor samples *CAV1* was more consistently methylated (Supplementary Table S2). This suggests that *CAV1* promoter methylation and ETS transcription factors do not cooperate in the regulation of *CAV1* expression in PCa. Although *IGFBP3* also showed a greater underexpression in PCa oETS+ when compared to PCa ERG+, there was a non-significant increase in *IGFBP3* expression after DAC treatment of the *ETV1-*positive LNCaP cell line, precluding a consistent relationship between higher *IGFBP3* methylation levels and *ETV1* rearrangements. We also confirmed that *TGFBR2* expression is reduced in PCa [50]–[51], which is compatible with the tumor suppressor role of *TGFBR2* in PCa cells described by others [52], but promoter methylation does not seem to be involved. On the other hand, we found that *NR0B1* was poorly expressed in PCa and in NPT, so our data do not support the previously reported immunoreactivity of DAX1 (protein encoded by the *NR0B1* gene) in a significant proportion of PCa [53].

Based on our microarray findings of differential expression of *ECRG4*, *LDOC1*, *HIST1H4L* and *KCNN2* between PCa harboring *ERG* rearrangements and those without ETS fusions, we decided to validate these data in an independent series of tumors. Among the five genes downregulated in PCa *ERG+*, we choose *ECRG4* and *LDOC1* for further study based on their tumor suppressor activity in other cancer models (see below; [43]–[44]). We also evaluated the expression of these genes in ESFT and ARMS in order to verify if there was any significant difference in their expression that might be attributable to *EWSR1*-ETS rearrangements. We here report for the first time that expression of both *ECRG4* and *LDOC1* is significantly decreased in PCa when compared to NPT. However, this was independent of the ETS status, contrarily to our initial microarray data suggesting a specific underexpression in PCa with *ERG* fusion genes. Consistent with a recent study that has associated CpG island hypermethylation of *ECRG4* with recurrence in prostate carcinoma [54], our MSP analysis showed a significantly higher methylation frequency in PCa comparing with NPT, thus representing a mechanism of gene silencing that might be involved in all molecular subgroups of PCa. In LNCaP and 22Rv1 cell lines, however, DAC treatment was not sufficient to allow *de novo ECRG4* expression, thus suggesting that other regulatory mechanisms may act in *ECRG4* underexpression. The mechanism of *LDOC1* downregulation is currently unknown, but because we did not find aberrant promoter methylation at this locus, other epigenetic or genetic alterations are probably causally involved. Finally, although the chimeric EWSR1-FLI1 protein has been found to bind the promoter of both *LDOC1* and *ECRG4 in vitro* [20], we here show that their expression is not significantly different between ESFT and ARMS, thus suggesting that either the expression of these genes is not regulated by that chimeric protein in ESFT or that a different regulatory mechanism in ARMS is regulating the expression of *LDOC1* and *ECRG4* to similar levels.

Our microarray findings of differential expression of *HIST1H4L* and *KCNN2* in different molecular subsets of PCa were confirmed by qRT-PCR in an independent series. *HIST1H4L* is a gene that encodes a histone, which is a basic nuclear protein responsible for the nucleosome structure of the chromosomal fiber in eukaryotes. We here show for the first time that *HIST1H4L* expression is specifically and significantly increased in PCa harboring *ERG* fusion genes, both when compared to other PCa molecular subtypes and with NPT. These findings indicate that *HIST1H4L* is a potential target of *ERG* fusion genes, as also illustrated by our demonstration of direct binding of ERG to the *HIST1H4L* promoter, but the mechanism whereby it is involved in prostate carcinogenesis is still unknown. *KCNN2* codes for a small conductance Ca^2+^ *-*activated potassium channel involved in the regulation of the neuronal excitability [55], and, to our knowledge, we here show for the first time that this gene is overexpressed in PCa harboring *ERG* rearrangements when compared to the other subtypes of PCa and to NPT. On the other hand, *KCNN2* was underexpressed in both PCa with other ETS rearrangements and in those without ETS rearrangements when compared to NPT. These data suggest that *KCNN2* regulation may be mediated by the aberrant *ERG* transcription factor in a particular subtype of PCa (PCa *ERG+*), as also illustrated by our demonstration of direct binding of ERG to the *KCNN2* promoter, and that different ETS can have specific roles, even in the same cellular context. Conversely, we show for the first time that *KCNN2* is significantly underexpressed in ESFT when compared to ARMS. Since it has been previously shown that EWSR1-FLI1 binds to the promoter of *KCNN2 in vitro* [20], it seems reasonable to assume that this downregulation of *KCNN2* in ESFT might be directly mediated by the chimeric transcription factor. On the other hand, although *HIST1H4L* was also found as a direct target of the EWSR1-FLI1 chimeric protein [20], our data showed that the expression of *HIST1H4L* was not significantly different between the ESFT and ARMS, thus suggesting that either EWSR1-FLI1 does not regulate *HIST1H4L* expression *in vivo* or that other regulatory mechanism in ARMS is regulating *HIST1H4L* to similar expression levels.

In conclusion, using two different models of ETS-related tumors, we show that, despite of the conservation of the DNA binding domain of the ETS family of transcription factors, ETS proteins can modulate common target genes in different manners, as well as achieve specificity by controlling distinct genes.

## Supporting Information

Figure S1 **Comparative methylation and expression levels of** ***CAV1*** **after DAC treatment of LNCaP and 22Rv1 prostate cancer cell lines.**
(TIF)

Table S1 **Assay ID or sequence of the primers used in this study.**
(DOC)

Table S2 **MSP analysis data of prostate samples.**
(DOC)

## References

[1] Nunn MF, Seeburg PH, Moscovici C, Duesberg PH (1983) Tripartite structure of the avian erythroblastosis virus E26 transforming gene. Nature 306: 391–395. 6316155 10.1038/306391a0

[2] Seth A, Watson DK (2005) ETS transcription factors and their emerging roles in human cancer. Eur J Cancer 41: 2462–2478. 16213704 10.1016/j.ejca.2005.08.013

[3] Sementchenko VI, Watson DK (2000) Ets target genes: past, present and future. Oncogene 19: 6533–6548. 11175369 10.1038/sj.onc.1204034

[4] Oikawa T, Yamada T (2003) Molecular biology of the Ets family of transcription factors. Gene 303: 11–34. 12559563 10.1016/s0378-1119(02)01156-3

[5] Graves BJ, Petersen JM (1998) Specificity within the ets family of transcription factors. Adv Cancer Res 75: 1–55. 9709806 10.1016/s0065-230x(08)60738-1

[6] Sharrocks AD (2001) The ETS-domain transcription factor family. Nat Rev Mol Cell Biol 2: 827–837. 11715049 10.1038/35099076

[7] Riggi N, Stamenkovic I (2007) The biology of Ewing sarcoma. Cancer Lett 254: 1–10. 17250957 10.1016/j.canlet.2006.12.009

[8] Tomlins SA, Rhodes DR, Perner S, Dhanasekaran SM, Mehra R, et al (2005) Recurrent fusion of TMPRSS2 and ETS transcription factor genes in prostate cancer. Science 310: 644–648. 16254181 10.1126/science.1117679

[9] Cerveira N, Ribeiro FR, Peixoto A, Costa V, Henrique R, et al (2006) TMPRSS2-ERG gene fusion causing ERG overexpression precedes chromosome copy number changes in prostate carcinomas and paired HGPIN lesions. Neoplasia 8: 826–832. 17032499 10.1593/neo.06427 PMC1715930

[10] Mehra R, Tomlins SA, Shen R, Nadeem O, Wang L, et al (2007) Comprehensive assessment of TMPRSS2 and ETS family gene aberrations in clinically localized prostate cancer. Mod Pathol 20: 538–544. 17334343 10.1038/modpathol.3800769

[11] Perner S, Demichelis F, Beroukhim R, Schmidt FH, Mosquera JM, et al (2006) TMPRSS2:ERG fusion-associated deletions provide insight into the heterogeneity of prostate cancer. Cancer Res 66: 8337–8341. 16951139 10.1158/0008-5472.CAN-06-1482

[12] Clark JP, Cooper CS (2009) ETS gene fusions in prostate cancer. Nat Rev Urol 6: 429–439. 19657377 10.1038/nrurol.2009.127

[13] Paulo P, Barros-Silva JD, Ribeiro FR, Ramalho-Carvalho J, Jeronimo C, et al (2012) FLI1 is a novel ETS transcription factor involved in gene fusions in prostate cancer. Genes Chromosomes Cancer 51: 240–249. 22081504 10.1002/gcc.20948

[14] Pflueger D, Rickman DS, Sboner A, Perner S, LaFargue CJ, et al (2009) N-myc downstream regulated gene 1 (NDRG1) is fused to ERG in prostate cancer. Neoplasia 11: 804–811. 19649210 10.1593/neo.09572 PMC2713587

[15] Chansky HA, Barahmand-Pour F, Mei Q, Kahn-Farooqi W, Zielinska-Kwiatkowska A, et al (2004) Targeting of EWS/FLI-1 by RNA interference attenuates the tumor phenotype of Ewing’s sarcoma cells in vitro. J Orthop Res 22: 910–917. 15183454 10.1016/j.orthres.2003.12.008

[16] Prieur A, Tirode F, Cohen P, Delattre O (2004) EWS/FLI-1 silencing and gene profiling of Ewing cells reveal downstream oncogenic pathways and a crucial role for repression of insulin-like growth factor binding protein 3. Mol Cell Biol 24: 7275–7283. 15282325 10.1128/MCB.24.16.7275-7283.2004 PMC479730

[17] Tomlins SA, Laxman B, Dhanasekaran SM, Helgeson BE, Cao X, et al (2007) Distinct classes of chromosomal rearrangements create oncogenic ETS gene fusions in prostate cancer. Nature 448: 595–599. 17671502 10.1038/nature06024

[18] Han B, Mehra R, Dhanasekaran SM, Yu J, Menon A, et al (2008) A fluorescence in situ hybridization screen for E26 transformation-specific aberrations: identification of DDX5-ETV4 fusion protein in prostate cancer. Cancer Res 68: 7629–7637. 18794152 10.1158/0008-5472.CAN-08-2014 PMC2760292

[19] Gangwal K, Lessnick SL (2008) Microsatellites are EWS/FLI response elements: genomic “junk” is EWS/FLI’s treasure. Cell Cycle 7: 3127–3132. 18927503 10.4161/cc.7.20.6892

[20] Gangwal K, Sankar S, Hollenhorst PC, Kinsey M, Haroldsen SC, et al (2008) Microsatellites as EWS/FLI response elements in Ewing’s sarcoma. Proc Natl Acad Sci U S A 105: 10149–10154. 18626011 10.1073/pnas.0801073105 PMC2481306

[21] Iljin K, Wolf M, Edgren H, Gupta S, Kilpinen S, et al (2006) TMPRSS2 fusions with oncogenic ETS factors in prostate cancer involve unbalanced genomic rearrangements and are associated with HDAC1 and epigenetic reprogramming. Cancer Res 66: 10242–10246. 17079440 10.1158/0008-5472.CAN-06-1986

[22] Tomlins SA, Laxman B, Varambally S, Cao X, Yu J, et al (2008) Role of the TMPRSS2-ERG gene fusion in prostate cancer. Neoplasia 10: 177–188. 18283340 10.1593/neo.07822 PMC2244693

[23] Hollenhorst PC, McIntosh LP, Graves BJ (2011) Genomic and biochemical insights into the specificity of ETS transcription factors. Annu Rev Biochem 80: 437–471. 21548782 10.1146/annurev.biochem.79.081507.103945 PMC5568663

[24] Laudet V, Hanni C, Stehelin D, Duterque-Coquillaud M (1999) Molecular phylogeny of the ETS gene family. Oncogene 18: 1351–1359. 10022817 10.1038/sj.onc.1202444

[25] Ribeiro FR, Paulo P, Costa VL, Barros-Silva JD, Ramalho-Carvalho J, et al (2011) Cysteine-rich secretory protein-3 (CRISP3) is strongly up-regulated in prostate carcinomas with the TMPRSS2-ERG fusion gene. PLoS ONE 6: e22317. 21814574 10.1371/journal.pone.0022317 PMC3141037

[26] Tirado OM, Mateo-Lozano S, Villar J, Dettin LE, Llort A, et al (2006) Caveolin-1 (CAV1) is a target of EWS/FLI-1 and a key determinant of the oncogenic phenotype and tumorigenicity of Ewing’s sarcoma cells. Cancer Res 66: 9937–9947. 17047056 10.1158/0008-5472.CAN-06-0927

[27] Mendiola M, Carrillo J, Garcia E, Lalli E, Hernandez T, et al (2006) The orphan nuclear receptor DAX1 is up-regulated by the EWS/FLI1 oncoprotein and is highly expressed in Ewing tumors. Int J Cancer 118: 1381–1389. 16206264 10.1002/ijc.21578

[28] Hahm KB, Cho K, Lee C, Im YH, Chang J, et al (1999) Repression of the gene encoding the TGF-beta type II receptor is a major target of the EWS-FLI1 oncoprotein. Nat Genet 23: 222–227. 10508522 10.1038/13854

[29] Meier VS, Kuhne T, Jundt G, Gudat F (1998) Molecular diagnosis of Ewing tumors: improved detection of EWS-FLI-1 and EWS-ERG chimeric transcripts and rapid determination of exon combinations. Diagn Mol Pathol 7: 29–35. 9646032 10.1097/00019606-199802000-00006

[30] Anderson J, Gordon A, Pritchard-Jones K, Shipley J (1999) Genes, chromosomes, and rhabdomyosarcoma. Genes Chromosomes Cancer 26: 275–285. 10534762

[31] Cerveira N, Torres L, Ribeiro FR, Henrique R, Pinto A, et al (2005) Multimodal genetic diagnosis of solid variant alveolar rhabdomyosarcoma. Cancer Genet Cytogenet 163: 138–143. 16337856 10.1016/j.cancergencyto.2005.06.020

[32] Pearson H, Stirling D (2003) DNA extraction from tissue. Methods Mol Biol 226: 33–34. 12958476 10.1385/1-59259-384-4:33

[33] Paulo P, Ribeiro FR, Santos J, Mesquita D, Almeida M, et al (2012) Molecular subtyping of primary prostate cancer reveals specific and shared target genes of different ETS rearrangements. Neoplasia 14: 600–611. 22904677 10.1593/neo.12600 PMC3421956

[34] Schmittgen TD, Livak KJ (2008) Analyzing real-time PCR data by the comparative C(T) method. Nat Protoc 3: 1101–1108. 18546601 10.1038/nprot.2008.73

[35] Takai D, Jones PA (2002) Comprehensive analysis of CpG islands in human chromosomes 21 and 22. Proc Natl Acad Sci U S A 99: 3740–3745. 11891299 10.1073/pnas.052410099 PMC122594

[36] Yamashita S, Tsujino Y, Moriguchi K, Tatematsu M, Ushijima T (2006) Chemical genomic screening for methylation-silenced genes in gastric cancer cell lines using 5-aza-2′-deoxycytidine treatment and oligonucleotide microarray. Cancer Sci 97: 64–71. 16367923 10.1111/j.1349-7006.2006.00136.x PMC11159443

[37] Sato N, Fukushima N, Maitra A, Matsubayashi H, Yeo CJ, et al (2003) Discovery of novel targets for aberrant methylation in pancreatic carcinoma using high-throughput microarrays. Cancer Res 63: 3735–3742. 12839967

[38] Ibanez de Caceres I, Cortes-Sempere M, Moratilla C, Machado-Pinilla R, Rodriguez-Fanjul V, et al (2010) IGFBP-3 hypermethylation-derived deficiency mediates cisplatin resistance in non-small-cell lung cancer. Oncogene 29: 1681–1690. 20023704 10.1038/onc.2009.454

[39] Barros R, da Costa LT, Pinto-de-Sousa J, Duluc I, Freund JN, et al (2011) CDX2 autoregulation in human intestinal metaplasia of the stomach: impact on the stability of the phenotype. Gut 60: 290–298. 21148572 10.1136/gut.2010.222323 PMC3034084

[40] Sandelin A, Wasserman WW, Lenhard B (2004) ConSite: web-based prediction of regulatory elements using cross-species comparison. Nucleic Acids Res 32: W249–252. 15215389 10.1093/nar/gkh372 PMC441510

[41] Wei GH, Badis G, Berger MF, Kivioja T, Palin K, et al (2010) Genome-wide analysis of ETS-family DNA-binding in vitro and in vivo. EMBO J 29: 2147–2160. 20517297 10.1038/emboj.2010.106 PMC2905244

[42] Massie CE, Mills IG (2011) Global identification of androgen response elements. Methods Mol Biol 776: 255–273. 21796531 10.1007/978-1-61779-243-4_15

[43] Li LW, Yu XY, Yang Y, Zhang CP, Guo LP, et al (2009) Expression of esophageal cancer related gene 4 (ECRG4), a novel tumor suppressor gene, in esophageal cancer and its inhibitory effect on the tumor growth in vitro and in vivo. Int J Cancer 125: 1505–1513. 19521989 10.1002/ijc.24513

[44] Nagasaki K, Schem C, von Kaisenberg C, Biallek M, Rosel F, et al (2003) Leucine-zipper protein, LDOC1, inhibits NF-kappaB activation and sensitizes pancreatic cancer cells to apoptosis. Int J Cancer 105: 454–458. 12712434 10.1002/ijc.11122

[45] Janknecht R (2005) EWS-ETS oncoproteins: the linchpins of Ewing tumors. Gene 363: 1–14. 16202544 10.1016/j.gene.2005.08.007

[46] Mao X, Miesfeldt S, Yang H, Leiden JM, Thompson CB (1994) The FLI-1 and chimeric EWS-FLI-1 oncoproteins display similar DNA binding specificities. J Biol Chem 269: 18216–18222. 7517940

[47] May WA, Lessnick SL, Braun BS, Klemsz M, Lewis BC, et al (1993) The Ewing’s sarcoma EWS/FLI-1 fusion gene encodes a more potent transcriptional activator and is a more powerful transforming gene than FLI-1. Mol Cell Biol 13: 7393–7398. 8246959 10.1128/mcb.13.12.7393-7398.1993 PMC364810

[48] Patel M, Simon JM, Iglesia MD, Wu SB, McFadden AW, et al (2012) Tumor-specific retargeting of an oncogenic transcription factor chimera results in dysregulation of chromatin and transcription. Genome Res 22: 259–270. 22086061 10.1101/gr.125666.111 PMC3266033

[49] Cui J, Rohr LR, Swanson G, Speights VO, Maxwell T, et al (2001) Hypermethylation of the caveolin-1 gene promoter in prostate cancer. Prostate 46: 249–256. 11170154 10.1002/1097-0045(20010215)46:3<249::aid-pros1030>3.0.co;2-#

[50] Guo Y, Jacobs SC, Kyprianou N (1997) Down-regulation of protein and mRNA expression for transforming growth factor-beta (TGF-beta1) type I and type II receptors in human prostate cancer. Int J Cancer 71: 573–579. 9178810 10.1002/(sici)1097-0215(19970516)71:4<573::aid-ijc11>3.0.co;2-d

[51] Williams RH, Stapleton AM, Yang G, Truong LD, Rogers E, et al (1996) Reduced levels of transforming growth factor beta receptor type II in human prostate cancer: an immunohistochemical study. Clin Cancer Res 2: 635–640. 9816213

[52] Danielpour D (2005) Functions and regulation of transforming growth factor-beta (TGF-beta) in the prostate. Eur J Cancer 41: 846–857. 15808954 10.1016/j.ejca.2004.12.027

[53] Nakamura Y, Suzuki T, Arai Y, Sasano H (2009) Nuclear receptor DAX1 in human prostate cancer: a novel independent biological modulator. Endocr J 56: 39–44. 18827407 10.1507/endocrj.k08e-177

[54] Vanaja DK, Ehrich M, Van den Boom D, Cheville JC, Karnes RJ, et al (2009) Hypermethylation of genes for diagnosis and risk stratification of prostate cancer. Cancer Invest 27: 549–560. 19229700 10.1080/07357900802620794 PMC2693083

[55] Szatanik M, Vibert N, Vassias I, Guenet JL, Eugene D, et al (2008) Behavioral effects of a deletion in Kcnn2, the gene encoding the SK2 subunit of small-conductance Ca2+-activated K+ channels. Neurogenetics 9: 237–248. 18604572 10.1007/s10048-008-0136-2

