# Supplementary figures and images for: Potential Downstream Target Genes of Aberrant ETS Transcription Factors Are Differentially Affected in Ewing’s Sarcoma and Prostate Carcinoma
Source: PLoS One. 2012 Nov 19;7(11):e49819. doi: 10.1371/journal.pone.0049819 (PMC3501462; doi:10.1371/journal.pone.0049819)

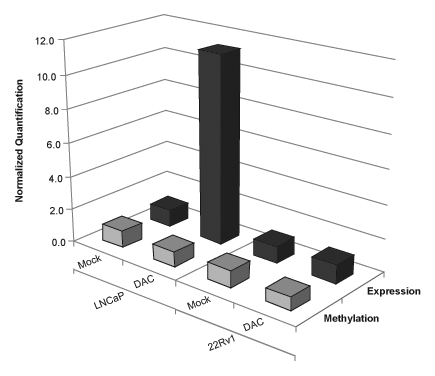

Supplement: Figure S1 — Comparative methylation and expression levels of CAV1 after DAC treatment of LNCaP and 22Rv1 prostate cancer cell lines. (TIF) [file pone.0049819.s001.tif]
