# Supplementary material for: Potential Downstream Target Genes of Aberrant ETS Transcription Factors Are Differentially Affected in Ewing’s Sarcoma and Prostate Carcinoma
Source: PLoS One. 2012 Nov 19;7(11):e49819. doi: 10.1371/journal.pone.0049819 (PMC3501462; doi:10.1371/journal.pone.0049819)
Supplement: Table S1 — Assay ID or sequence of the primers used in this study. (DOC) [file pone.0049819.s002.doc]

**Supplementary Table S1.** Assay ID or sequence of the primers used in this study.

| Assay | Assay/Primer Name | Assay ID/Primer sequence 5’-3’ |
| --- | --- | --- |
| **qRT-PCR** | CAV1 | Hs00971716_m1 |
| **qRT-PCR** | NR0B1 | Hs00230864_m1 |
| **qRT-PCR** | IGFBP3 | Hs00426289_m1 |
| **qRT-PCR** | TGFBR2 | Hs00559661_m1 |
| **qRT-PCR** | HIST1H4L | Hs00361930_s1 |
| **qRT-PCR** | KCNN2 | Hs01030641_m1 |
| **qRT-PCR** | ECRG4 | Hs00963149_m1 |
| **qRT-PCR** | LDOC1 | Hs00950598_s1 |
| **qRT-PCR** | GUSB | 4333767F |
| **MSP** | CAV1-F | TTTCGGGACGTTTTTCGGTGGT |
| **MSP** | CAV1-R | TAAAAACGTTTCTCCCGCGCTA |
| **MSP** | IGFBP3-F | TTTTACGAGGTATATACGAATGC |
| **MSP** | IGFBP3-R | TCTCGAAATAAAATCTCCCTACG |
| **MSP** | TGFBR2-F | TCGGTTTATGACGAGTAGC |
| **MSP** | TGFBR2-R | AAAATCGTACTAACGATACGC |
| **MSP** | ECRG4-F | AGGATTTCGGTGGTATTC |
| **MSP** | ECRG4-R | GCGAATTATCCCTACGTC |
| **MSP** | LDOC1-F | TATTAAGCGTTTTCGTGGATAC |
| **MSP** | LDOC1-R | CGACCTAAAAAAACAAAACTCG |
| **ChIP-qPCR** | pHIST1H4L-454F | TGCAGTCAGCCACTCCATAG |
| **ChIP-qPCR** | pHIST1H4L-454R | TTGCAGCCTGTTTTTCCTGT |
| **ChIP-qPCR** | pHIST1H4L-728F | AGGAACATGAGGGAGCTCAA |
| **ChIP-qPCR** | pHIST1H4L-728R | TCCACCTAGAAACACTTCAGGA |
| **ChIP-qPCR** | pHIST1H4L-2266F | TTAGTAGGGACCGGATGTGG |
| **ChIP-qPCR** | pHIST1H4L-2266R | AAGGCTGGAGTGCGATCTT |
| **ChIP-qPCR** | pKCNN2-1442-F | CACCCAATCCAGATTCCAGAG |
| **ChIP-qPCR** | pKCNN2-1442-R | CTTTGGGGAGAAAAAGTTTGG |
| **ChIP-qPCR** | pKCNN2-1833-F | AGGTGAGGAGGAGGAAGAGAAG |
| **ChIP-qPCR** | pKCNN2-1833-R | CTCCAGAACATGAGGGGATG |
| **ChIP-qPCR** | pKCNN2-4083F | TGGCTGAGTATGACCTCTGG |
| **ChIP-qPCR** | pKCNN2-4083R | GGAGAGAGGAAGGAAATGAAGA |
| **ChIP-qPCR** | Control-F | TGCCTCAGATTTGGAGTGCT |
| **ChIP-qPCR** | Control-R | GAGAAGCCTCTGAGGAGGGA |
| **F-*forward*; R-*reverse*** | | |
