# Supplementary material for: Potential Downstream Target Genes of Aberrant ETS Transcription Factors Are Differentially Affected in Ewing’s Sarcoma and Prostate Carcinoma
Source: PLoS One. 2012 Nov 19;7(11):e49819. doi: 10.1371/journal.pone.0049819 (PMC3501462; doi:10.1371/journal.pone.0049819)
Supplement: Table S2 — MSP analysis data of prostate samples. (DOC) [file pone.0049819.s003.doc]

**Supplementary Table S2.** MSP analysis data of prostate samples.

|  |  | **Promoter Methylation Status** | | | | |
| --- | --- | --- | --- | --- | --- | --- |
| **Prostate Sample** | **ETS Status** | ***CAV1*** | ***IGFBP3*** | ***TGFBR2*** | ***ECRG4*** | ***LDOC1*** |
| NPT1 | NA | Negative | Negative | ND | Negative | ND |
| NPT5 | NA | ND | Negative | ND | Positive | ND |
| NPT6 | NA | Negative | Negative | ND | Negative | ND |
| NPT7 | NA | ND | Negative | ND | Negative | ND |
| NPT8 | NA | ND | Negative | ND | Negative | ND |
| NPT9 | NA | ND | Negative | ND | Negative | ND |
| NPT10 | NA | Negative | Negative | ND | Negative | ND |
| NPT11 | NA | ND | Negative | ND | Positive | ND |
| NPT12 | NA | Negative | Negative | ND | Negative | ND |
| NPT13 | NA | ND | Negative | ND | Negative | ND |
| NPT14 | NA | Negative | Negative | ND | Negative | ND |
| NPT20 | NA | ND | Negative | ND | Negative | ND |
| NPT21 | NA | ND | Negative | ND | Negative | ND |
| NPT23 | NA | Negative | Negative | ND | Negative | ND |
| NPT26 | NA | Negative | Negative | ND | Negative | ND |
| PCa-209T | ERG+ | ND | Positive | Negative | Negative | ND |
| PCa-227T | ERG+ | Positive | ND | ND | ND | ND |
| PCa-238T | ERG+ | ND | ND | Negative | Negative | Negative |
| PCa-254T | ERG+ | ND | ND | Negative | Positive | ND |
| PCa-265T | ERG+ | ND | ND | ND | Positive | Negative |
| PCa-276T | ERG+ | ND | ND | ND | Positive | ND |
| PCa-288T | ERG+ | Positive | ND | ND | Positive | ND |
| PCa-307T | ERG+ | ND | ND | ND | ND | Negative |
| PCa-229T | ETS- | Positive | Positive | ND | ND | ND |
| PCa-242T | ETS- | Positive | ND | ND | ND | Negative |
| PCa-279T | ETS- | ND | ND | Negative | Positive | Negative |
| PCa-281T | ETS- | ND | ND | ND | Negative | ND |
| PCa-313T | ETS- | Positive | ND | ND | ND | ND |
| PCa-340T | ETS- | ND | Negative | Negative | Positive | Negative |
| PCa-470T | ETS- | ND | Negative | ND | Negative | ND |
| PCa-262T | oETS+(ETV1) | Negative | Positive | ND | Positive | Negative |
| PCa-272T | oETS+(ETV1) | ND | ND | Negative | Negative | ND |
| PCa-274T | oETS+(ETV1) | Negative | ND | ND | ND | ND |
| PCa-305T | oETS+(ETV1) | Positive | ND | ND | ND | ND |
| PCa-488T | oETS+(ETV1) | ND | Positive | ND | ND | ND |
| PCa-499T | oETS+(ETV1) | Positive | Positive | ND | Positive | ND |
| PCa-525T | oETS+(ETV1) | ND | Positive | Negative | Positive | Negative |
| PCa-542T | oETS+(ETV1) | Negative | ND | ND | ND | ND |
| NA- not applicable; ND- not done | | | | | | |
